# Performance of a Deep Learning System for Automatic Diagnosis of Protruding Lesions in Colon Capsule Endoscopy

**DOI:** 10.3390/diagnostics12061445

**Published:** 2022-06-12

**Authors:** Miguel Mascarenhas, João Afonso, Tiago Ribeiro, Hélder Cardoso, Patrícia Andrade, João P. S. Ferreira, Miguel Mascarenhas Saraiva, Guilherme Macedo

**Affiliations:** 1 Department of Gastroenterology, São João University Hospital, Alameda Professor Hernâni Monteiro, 4200-427 Porto, Portugal; joaoafonso28@gmail.com (J.A.); tiagofcribeiro@outlook.pt (T.R.); hc@sapo.pt (H.C.); anapatriciarandrade@gmail.com (P.A.); guilhermemacedo59@gmail.com (G.M.); 2 WGO Gastroenterology and Hepatology Training Center, 4200-427 Porto, Portugal; 3 Faculty of Medicine of the University of Porto, Alameda Professor Hernâni Monteiro, 4200-427 Porto, Portugal; 4 Department of Mechanical Engineering, Faculty of Engineering of the University of Porto, Rua Dr. Roberto Frias, 4200-465 Porto, Portugal; j.ferreira@fe.up.pt; 5 INEGI—Institute of Science and Innovation in Mechanical and Industrial Engineering, Rua Dr. Roberto Frias, 4200-465 Porto, Portugal; 6 ManopH Gastroenterology Clinic, Rua Sá da Bandeira 752, 4000-432 Porto, Portugal; miguelms.manoph@gmail.com

**Keywords:** colon capsule endoscopy, artificial intelligence, convolutional neural network, colorectal neoplasia

## Abstract

Background: Colon capsule endoscopy (CCE) is an alternative for patients unwilling or with contraindications for conventional colonoscopy. Colorectal cancer screening may benefit greatly from widespread acceptance of a non-invasive tool such as CCE. However, reviewing CCE exams is a time-consuming process, with risk of overlooking important lesions. We aimed to develop an artificial intelligence (AI) algorithm using a convolutional neural network (CNN) architecture for automatic detection of colonic protruding lesions in CCE images. An anonymized database of CCE images collected from a total of 124 patients was used. This database included images of patients with colonic protruding lesions or patients with normal colonic mucosa or with other pathologic findings. A total of 5715 images were extracted for CNN development. Two image datasets were created and used for training and validation of the CNN. The AUROC for detection of protruding lesions was 0.99. The sensitivity, specificity, PPV and NPV were 90.0%, 99.1%, 98.6% and 93.2%, respectively. The overall accuracy of the network was 95.3%. The developed deep learning algorithm accurately detected protruding lesions in CCE images. The introduction of AI technology to CCE may increase its diagnostic accuracy and acceptance for screening of colorectal neoplasia.

## 1. Introduction

Capsule endoscopy (CE) is a primary diagnostic tool for the investigation of patients with suspected small bowel disease. Colon capsule endoscopy has been recently introduced as a minimally invasive alternative to conventional colonoscopy for evaluation of the colonic mucosa [1,2]. This system allows overcoming some of the drawbacks associated with conventional colonoscopy, including the potential for pain, use of sedation, and the risk of adverse events, including bleeding and perforation [3]. The clinical application of this tool has been most extensively studied in the setting of colorectal cancer screening, particularly for patients with previous incomplete colonoscopy, or for whom the latter exam is contraindicated, unfeasible or unwanted [4,5]. The role of CCE as an alternative to conventional colonoscopy in the setting of colorectal cancer screening is growing. A recent meta-analysis by Vuik and coworkers reported similar performance levels for conventional colonoscopy and CCE as well as the superiority of CCE compared to computed tomography colonography (virtual colonoscopy) [6]. Moreover, a single full-length CCE video may produce over 50,000 images, and reviewing these images is a monotonous and time-consuming task, requiring approximately 50 min for completion [2]. Furthermore, any given frame may capture only a fragment of a mucosal abnormality and lesions may be depicted in a very small number of frames. Therefore, the risk of overlooking important lesions is not insignificant [2].

The combination of enhanced computational power with large clinical datasets has potentiated the research and development of AI tools for clinical implementation. The application of automated algorithms to diverse medical fields has provided promising results regarding disease identification and classification [7,8,9]. Convolutional neural networks (CNN) are a type of multi-layered deep learning algorithm tailored for image analysis. The application of these technological solutions to small bowel CE has provided promising results in the detection of several types of lesions [10,11,12,13]. The introduction of AI tools for real-time detection of colorectal neoplasia in conventional colonoscopy has suggested a high diagnostic yield for CNN-based algorithms [14]. The impact of AI algorithms for detection of colorectal neoplasia in CCE images has been scarcely evaluated. Enhanced reading of CCE images through the application of these tools may improve the diagnostic accuracy of CCE for colorectal neoplasia, which is currently unsatisfactory [2]. Importantly, the implementation of automated algorithms may help to reduce the time required for reading a single CCE exam. The aim of this study was to develop and validate a CNN-based algorithm for the automatic detection of colonic protruding lesions using CCE images.

## 2. Materials and Methods

### 2.1. Study Design

A multicenter study was performed for development and validation of a CNN for automatic detection of colonic protruding lesions. CCE images were retrospectively collected from two different institutions: São João University Hospital (Porto, Portugal) and ManopH Gastroenterology Clinic (Porto, Portugal). One hundred and twenty-four CCE exams (124 patients, 24 from São João University Hospital and 100 from ManopH Gastroenterology Clinic), performed between 2010 and 2020, were included. The full-length video of all participants was reviewed, and a total of 5715 frames of the colonic mucosa were ultimately extracted. Significant frames were included regardless of image quality and bowel cleansing quality. Inclusion and classification of frames were performed by three gastroenterologists with experience in CCE (Miguel Mascarenhas, Hélder Cardoso and Miguel Mascarenhas Saraiva), each with an experience of >1500 CE previous to this study. A final decision on frame labelling required the agreement of at least two of the three researchers.

This study was approved by the ethics committee of São João University Hospital (No. CE 407/2020). The study protocol was conducted respecting the original and subsequent revisions of the declaration of Helsinki. This study is retrospective and of non-interventional nature. Thus, the output provided by the CNN had no influence on the clinical management of each included patient. Any information susceptible to identify the included patients was omitted, and each patient was assigned a random number in order to guarantee effective data anonymization for researchers involved in CNN development. A team with Data Protection Officer (DPO) certification (Maastricht University) confirmed the non-traceability of data and conformity with the general data protection regulation (GDPR).

#### 2.1.1. Colon Capsule Endoscopy Procedure

For all patients, CCE procedures were conducted using the *PillCam™* COLON 2 system (Medtronic, Minneapolis, MN, USA). This system consists of three major components: the endoscopic capsule, an array of sensors connected to a data recorder, and a software for frame revision. The capsule measures 32.3 mm in length and 11.6 mm in width. It has 2 high-resolution cameras, each with a 172° angle of view. The system frame rate varied automatically between 4 and 35 frames per second, depending on bowel motility. Each frame had a resolution of 512 × 512 pixels. The battery of the endoscopic capsule has an estimated life of ≥10 h [2]. This system was launched in 2009 and was not submitted to hardware updates since then. Thus, no significant changes in image quality were evident during this period. The images were reviewed using *PillCam™* software version 9.0 (Medtronic, Minneapolis, MN, USA). Each frame was processed in order to remove information allowing patient identification (name, operating number, date of procedure).

Each patient received bowel preparation according to previously published guidelines [15]. Summarily, patients initiated a clear liquid diet in the day preceding capsule ingestion, with fasting in the night before examination. A solution consisting of polyethylene glycol was used in split-dosage (2 L in the evening and 2 L in the morning of capsule ingestion). Prokinetic therapy (10 mg domperidone) was used if the capsule remained in the stomach 1 h after ingestion, upon real-time image review on the recorder. Two boosters consisting of a sodium phosphate solution were applied after the capsule has entered the small bowel and with a 3 h interval. Only complete CCE exams were included. A complete exam was considered if the capsule was excreted.

#### 2.1.2. Development of the Convolutional Neural Network

**Figure 1:**
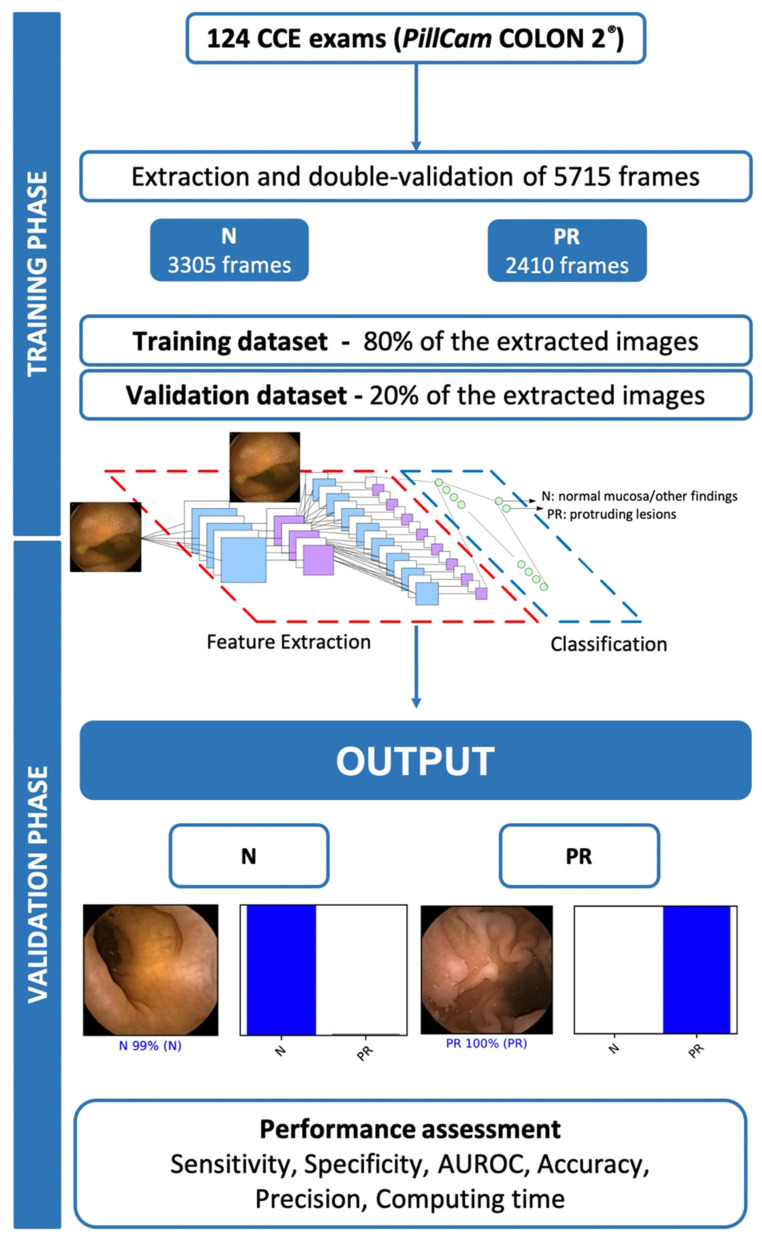
Summary of study design for the training and validation phases. PR—protruding lesion; N—normal mucosa or other findings.

A deep learning CNN was developed for automatic detection of colonic protruding lesions. Protruding lesions included all polyps, epithelial tumors, and subepithelial lesions. From the collected pool of images (*n* = 5715), 2410 showed protruding lesions and 3305 displayed normal mucosa or other mucosal lesions (ulcers, erosions, red spots, angiectasia, varices and lymphangiectasia). This pool of images was split for constitution of training and validation image datasets. The training dataset was composed by 80% of the consecutively extracted images (*n* = 4572). The remaining 20% were used as the validation dataset (*n* = 1143). The validation dataset was used for assessing the performance of the CNN (Figure 1).

To create the CNN, we modified the *Xception* model with its weights trained on *ImageNet* (a large-scale image dataset aimed for use in development of object recognition software). To transfer this learning to our data, we kept the convolutional layers of the model. We replaced the last fully connected layers with 2 dense layers of size 2048 and 1024, respectively, and then attached a fully connected layer based on the number of classes we used to classify our endoscopic images. To avoid overfitting, a dropout layer of 0.3 drop rate was added between convolutional and classification components of the network. We applied gradient-weighted class activation mapping on the last convolutional layer [16], in order to highlight important features for predicting protruding lesions. The size of each image was set for 300 pixels of height and width. The learning rate of 0.0001, batch size of 128 and the number of epochs of 30 was set by trial and error. We used Tensorflow 2.3 and Keras libraries to prepare the data and run the model. The analyses were performed with a computer equipped with a 2.1 GHz Intel^®^ Xeon^®^ Gold 6130 processor (Intel, Santa Clara, CA, USA) and a double NVIDIA Quadro^®^ RTX™ 8000 graphic processing unit (NVIDIA Corporate, Santa Clara, CA, USA).

#### 2.1.3. Model Performance and Statistical Analysis

**Figure 2:**
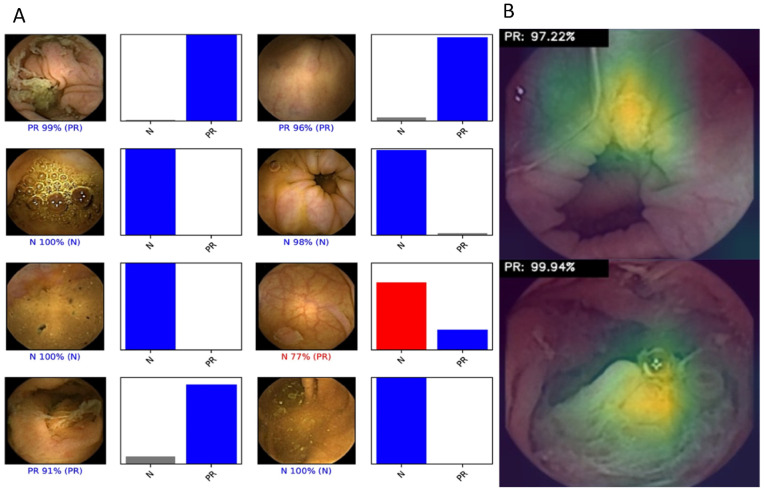
Heatmaps (**A**) and output (**B**) obtained from the application of the convolutional neural network. (**A**) Examples of heatmaps showing CCE features of protruding lesions as identified by the CNN. (**B**) The bars represent the probability estimated by the network.

The primary outcome measures included sensitivity, specificity, positive and negative predictive values, and accuracy. Moreover, we used receiver operating characteristic (ROC) curve analysis and area under the ROC curve (AUROC) to measure the performance of our model in the distinction between the categories. For each image, the trained CNN calculated the probability for each of the categories (protruding lesions vs. normal colonic mucosa or other findings). A higher probability value translated in a greater confidence in the CNN prediction. The software generated heatmaps that localized features that predicted a class probability (Figure 2A). The category with the highest probability score was outputted as the CNN’s predicted classification (Figure 2B). The output provided by the network was compared to the specialists’ classification (*gold standard*). We performed a 3-fold cross validation. Therefore, the entire dataset was split into 3 even-sized image groups. Training and validation datasets were created for each of the five groups, at a proportion of 80% and 20% for training and validation datasets, respectively. Sensitivities, specificities, positive and negative predictive values are presented as means ± standard deviations (SD). Additionally, the image processing performance of the network was determined by calculating the time required for the CNN to provide output for all images in the validation image dataset. Sensitivities, specificities, positive and negative predictive values were obtained using one iteration and are presented as percentages. Statistical analysis was performed using Sci-Kit learn v0.22.2 [17].

## 3. Results

### 3.1. Construction of the Network

**Figure 3:**
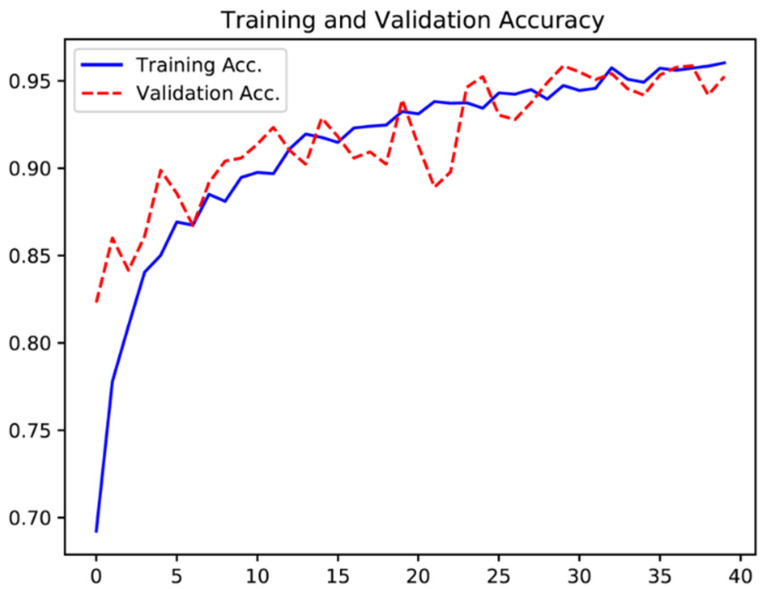
Evolution of the accuracy of the convolutional neural network during training and validation phases, as the training and validation datasets were repeatedly inputted in the neural network.

One hundred and twenty-four patients were submitted to CCE and enrolled in this study. A total of 5715 frames were extracted, 2410 showing protruding lesions (2303 polyps, 8 subepithelial lesions and 99 epithelial tumors) and 3305 showing normal colonic mucosa or other findings. The training dataset was constituted by 80% of the total image pool. The remaining 20% of frames (*n* = 1143) were used for testing the model. This validation dataset was composed by 482 (42.2%) images with evidence of protruding lesions and 661 (57.8%) images with normal colonic mucosa/other findings. The CNN evaluated each image and predicted a classification (protruding lesions vs. normal mucosa/other lesions), which was compared with the classification provided by gastroenterologists. Repeated inputs of data to the CNN resulted in the improvement of its accuracy (Figure 3).

### 3.2. Overall Performance of the Network

**Figure 4:**
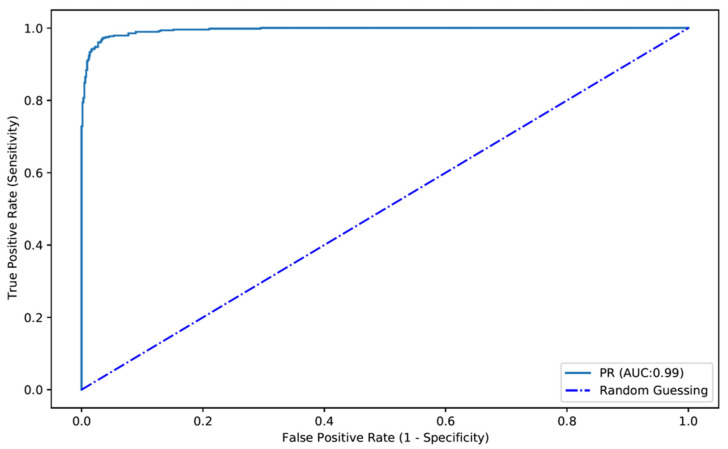
ROC analyses of the network’s performance in the detection of protruding lesions vs. normal colonic mucosa/other findings. ROC—receiver operator characteristics. PR—protruding lesion.

The confusion matrix between the trained CNN and expert classifications is shown in Table 1. Overall, the developed model had a sensitivity and specificity for the detection of protruding lesions of 90.0% and 99.1%, respectively. The positive and negative predictive values were, respectively, 98.6% and 93.2%. The overall accuracy of the network was 95.3% (Table 1). The AUROC for detection of protruding lesions was 0.99 (Figure 4).

We performed a 3-fold cross validation, where the entire dataset was randomized and split in 3 equivalent parts. The performance results for the three experiments are shown in Table 2. Overall, the estimated model accuracy was 95.6 ± 1.1%. The mean sensitivity and specificity of the model were 87.4 ± 4.6% and 96.1 ± 1.4%. The algorithm had a mean AUC of 0.976 ± 0.006.

### 3.3. Computational Performance of the CNN

The CNN completed the reading of the testing dataset in 17.5 s (approximately 15.4 ms/frame). This translates into an approximated reading rate of 65 frames per second. At this rate, the CNN would complete the revision of a full-length CCE video containing an estimate of 50,000 frames in approximately 13 min.

## 4. Discussion

The exploration of AI algorithms for application to conventional endoscopic techniques for automatic detection of colorectal neoplasia has been producing promising results over the last decade. The development and implementation of these systems has been recently endorsed (although with limitations) by the European Society of Gastrointestinal Endoscopy [18]. Furthermore, a recent meta-analysis has suggested that the application of AI models for adenoma and polyp’s identification may substantially increase the adenoma detection rate and the number of adenomas detected per colonoscopy [19]. These improvements in commonly used performance metrics have shown not to be affected by factors known to influence detection by the human eye, including the size and morphology of the lesions [19]. Artificial intelligence is expected to play a major role in improving the acceptability and the diagnostic yield of CCE [20]. These systems may help in several steps of the CCE process, from predicting the quality of colon cleanliness, lesion detection and the distinction of colorectal lesions [20,21,22].

In our study, we have developed a deep learning tool based on a CNN architecture for automatic detection of protruding lesions in the colonic lumen using CCE images. This study has several highlights. First, our model demonstrated high levels of performance, with a sensitivity of 90.0%, a specificity of 99.1, an accuracy of 95.3% and an AUROC of 0.99. Obtaining fairly high levels of sensitivity and negative predictive value is paramount for CNN-assisted reading systems, which are designed to lessen the probability of missing lesions, while maintaining a high specificity. Third, our network had a remarkable image processing performance, being capable of reading 65 images per second.

The precise role of CCE in everyday clinical practice is yet to be defined. Thus far, most studies highlight its potential when applied in the setting of colorectal cancer screening. Although colonoscopy remains the undisputed *gold standard*, studies have suggested that CCE could be viewed as a non-invasive complement, rather than substitutive of conventional colonoscopy, particularly in the setting of a previous incomplete colonoscopy [23]. Current guidelines on colorectal cancer screening list CCE as a valid alternative to colonoscopy for the screening of an average-risk population [15]. Studies comparing the diagnostic yield of CCE with another non-invasive screening test, CT colonography, have shown the superiority of CCE [24]. Moreover, when following a first positive fecal-immunological test, CCE may reduce the need for more invasive conventional colonoscopy [25]. Although conflicting evidence exist, some studies have shown that adoption of CCE as a screening method may lead to a higher uptake rate compared to conventional colonoscopy [26]. Moreover, CCE may not only be seen as an alternative to conventional colonoscopy but rather as a complementary solution in programmed screening settings. Indeed, CCE may help to shorten waiting lists, decrease hospital appointments and make screening available to remote areas [27]. In this setting, the cost-effectiveness of CCE appears to be greater when the prevalence of colorectal cancer is lower and the uptake rate is superior to that of conventional colonoscopy [28]. However, the use of CCE is hampered by its purely diagnostic character, the need for a rigorous bowel cleansing protocol, as well as the time required for reading each CCE exam.

The development of AI tools for detection of colorectal neoplasia in CCE images has been poorly explored. Automatic detection of these lesions is limited by the poor resolution of CCE images combined with their variable morphology, size and color. To our knowledge, only two other studies have assessed the potential of the application of CNN models to CCE images. Yamada et al. was the first to explore the implementation of AI algorithms for the identification of colorectal neoplasia in frames extracted from CCE exams. Their network was developed using a relatively large pool of CCE images (17,783 frames from 178 patients). Overall, their algorithm achieved a good performance (AUROC of 0.90) [29]. However, the sensitivity of their model was modest (79%) compared to that of our network. Blanes-Vidal et al. adapted a preexisting CNN (*AlexNet*) and trained it for the detection of colorectal polyps. The sensitivity, specificity and accuracy expressed in their paper were 97%, 93% and 96%, respectively. In our perspective, the development of these technologies should aim to support a clinical decision rather than substitute the role of the clinician. Therefore, these systems must remain highly sensitive in order to minimize the risk of missing lesions.

Our network demonstrated a high image processing performance (65 frames/second). To date, no value for comparison exists regarding CCE. Nevertheless, these performance marks exceed those published for CNNs applied to other CE systems [11,30]. The development of highly efficient networks may, in the near future, translate into shorter reading times, thus overcoming one of the main drawbacks of CCE. Further well-designed studies are required to assess if a high image processing capacity in experimental settings can be reproduced as an enhanced time efficiency regarding reading times of CCE exams comparing to conventional reading. The combination of enhanced diagnostic accuracy and time efficiency may have a pivotal role in widening the indications for CCE and its acceptance as a valid screening and diagnostic tool.

This study has several limitations. First, it is a retrospective study. Therefore, further prospective multicentric studies in a real-life setting are desirable to confirm the clinical value of our results. Second, although we included a large number of patients from two distinct medical centers, the number of extracted images is small. This limited number of extracted images was mainly dictated by the low number of frames showing protruding lesions. In order to produce a balanced dataset while minimizing the risk of missing lesions, an equilibrium between the number of images showing protruding lesions and normal mucosa was fostered. This may partially explain the suboptimal sensitivity. We are currently expanding our image pool in order to increase the robustness of our model, thus contributing to decrease the rate of false negative CE exams, which should be one of the main endpoints in developing these algorithms. The multicentric nature of our work reinforces the validity of our results. Nevertheless, multicentric studies including larger populations are required to ensure the clinical significance of our findings. Moreover, future studies for clinical validation of these tools must contemplate the comparison of performance between AI software and conventional colonoscopy, the *gold standard* technique for the detection and characterization of these lesions.

In conclusion, we developed a highly sensitive and specific CNN-based model for detection of protruding lesions in CCE images. We believe that the implementation of AI tools to clinical practice will be a crucial step for wider acceptance of CCE for non-invasive screening and diagnosis of colorectal neoplasia. Future studies should assess the impact of AI algorithms in mitigating the limitations of CCE in a real-life clinical setting, particularly the time required for reviewing each exam, as well as evaluate the potential benefits in terms of diagnostic yield.

## Figures and Tables

**Table 1:** Confusion matrix and performance marks.

		Expert Classification	
		Protruding Lesion	Normal Mucosa
CNN classification	Protruding lesion	434	6
	Normal mucosa	48	655
	Sensitivity	90.0%	
	Specificity	99.1%	
	PPV	98.6%	
	NPV	93.2%	
	Accuracy	95.3%	

Abbreviations: CNN—convolutional neural network; PPV—positive predictive value; NPV—negative predictive value.

**Table 2:** Three-fold cross validation.

	Sensitivity (%)	Specificity (%)	PPV (%)	NPV (%)	Accuracy (%)	AUC
**Fold 1**	82.8	97.5	62.6	99.1	96.9	0.980
**Fold 2**	87.4	95.9	57.1	99.2	95.4	0.970
**Fold 3**	92.1	94.7	48.4	99.6	94.6	0.980
**Overall, mean (±SD)**	87.4 ± 4.6	96.1 ± 1.4	56.0 ± 7.1	99.3 ± 0.2	95.6 ± 1.1	0.976 ± 0.006

Abbreviations: ±SD—±standard deviation; PPV—positive predictive value; NPV—negative predictive value; AUC—area under the receiving operator characteristics curve.
